# Photon-Counting Arrays for Time-Resolved Imaging

**DOI:** 10.3390/s16071005

**Published:** 2016-06-29

**Authors:** I. Michel Antolovic, Samuel Burri, Ron A. Hoebe, Yuki Maruyama, Claudio Bruschini, Edoardo Charbon

**Affiliations:** 1 Applied Quantum Architecture Lab (AQUA), Quantum Engineering Department, Delft University of Technology, Delft 2628CD, The Netherlands; i.m.antolovic@tudelft.nl; 2 Advanced Quantum Architecture lab (AQUA), Microengineering Department, Ecole Polytechnique Fédérale de Lausanne (EPFL), Lausanne 1015, Switzerland; samuel.burri@epfl.ch (S.B.); claudio.bruschini@epfl.ch (C.B.); 3 Academic Medical Centre, University of Amsterdam, Amsterdam 110DD, The Netherlands; r.a.hoebe@amc.uva.nl; 4 Jet Propulsion Lab, Pasadena, CA 91109, USA; y.maruyama@tudelft.nl

**Keywords:** single-photon avalanche diode, SPAD, fluorescence, fluorescence lifetime imaging microscopy, FLIM, fluorescence correlation spectroscopy, FCS

## Abstract

The paper presents a camera comprising 512 × 128 pixels capable of single-photon detection and gating with a maximum frame rate of 156 kfps. The photon capture is performed through a gated single-photon avalanche diode that generates a digital pulse upon photon detection and through a digital one-bit counter. Gray levels are obtained through multiple counting and accumulation, while time-resolved imaging is achieved through a 4-ns gating window controlled with subnanosecond accuracy by a field-programmable gate array. The sensor, which is equipped with microlenses to enhance its effective fill factor, was electro-optically characterized in terms of sensitivity and uniformity. Several examples of capture of fast events are shown to demonstrate the suitability of the approach.

## 1. Introduction

Photon counting and single-photon detection have been available at least since the 1930s, with the phtomultiplier tube (PMT) first and microchannel plate (MCP) later. These devices enable relatively high sensitivity, known in this context as photon detection efficiency (PDE), and low dark counts, quantified in terms of dark count rate (DCR), however they are generally bulky and they require high voltages to operate, typically hundreds to thousands of volts. In the 1940s, researchers started working with solid-state diodes operating in avalanche mode, known as avalanche photodiodes (APDs); these devices were refined through the 1950s and 1960s to be then implemented in planar processes. The devices required p-n junctions with guard rings and enhancement regions to prevent premature breakdown at the edge of the junction. With the improvement of semiconductor processes and the availability of more options, Cova and others began experimenting with Geiger-mode APDs or single-photon avalanche diodes (SPADs) in the 1970s and 1980s, recognizing the potential of these devices in capturing fast processes [1]. In the early 2000s, SPADs could be implemented in high-voltage processes first [2] and in standard CMOS image sensor processes later [3,4,5,6].

With the availability of SPADs in deep-submicron CMOS processes, it became conceivable to implement useful functionality in situ, possibly in pixel, so as to count photons and to time stamp them upon detection. The major consequence of this trend was massively parallel timestamping with picosecond resolution (LSB), with a possible explosion of data generated on chip [7]. Timestamping impinging photons individually has several uses. For instance, through time-correlated single-photon counting (TCSPC) [8], it becomes possible to accurately characterize photo responses of fluorophores when excited by fast light pulses. Fluorophores exhibit a time-dependent behavior, known as lifetime, that is specific to the fluorophore and/or the environment it is in [9]. Fluorescence lifetime imaging microscopy (FLIM) [10] is a technique used to characterize lifetime in fluorophores using multiple excitations and histogramming; in general, FLIM may be used in a confocal microscope with a single pixel, but it can also be used in widefield microscopy with a large number of pixels capable of performing TCSPC independently, thus speeding up lifetime capture by several orders of magnitude [11,12].

The first pixel with embedded time-to-digital converter (TDC) for in situ TCSPC was introduced in the project MEGAFRAME [13,14]. The drawback of this approach was the reduced fill factor that in turn required microlenses to recover, at least in part, lost sensitivity. As an alternative, researchers proposed to use simpler pixels, with a single digital counter [15] or with dual digital counters [16]. The use of analog counters was also proposed to ensure large resolution at low cost in terms of fill factor [17,18,19]. Due to the lack of a TDC though, these methods require a precise gate and significant algorithmic complexity [20,21].

In this paper, we describe a photon counting imager comprising a programmable global shutter with sub-150 ps skew and a minimum width of 4ns for time-resolved imaging applications. The image sensor comprises an array of 512 × 128 SPAD pixels that are read out in rolling mode, while the shutter itself is global. Since each pixel has a one-bit counter embedded in it, a frame is read as a binary matrix and can be converted to a multi-bit matrix externally by adding up subsequent frames, as first shown in [22]. The chip was demonstrated for fast fluorescence imaging and could be used for FLIM in [23]. Throughout the paper, significant attention was given to circuit details that led to the exceptional skew and to tradeoffs used during the design to achieve the target readout speed. A complete dynamic and static characterization of the chip was also provided with images exemplifying the suitability of the approach.

The paper is organized as follows: Section 2 describes the architecture of the sensor and its components. Section 3 analyzes the implications of using binary pixels towards image quality and Section 4 and Section 5 report the optical and electrical characterization of the sensor in the context of the target applications. Section 6 concludes the paper.

## 2. Sensor Architecture

**Figure 1:**
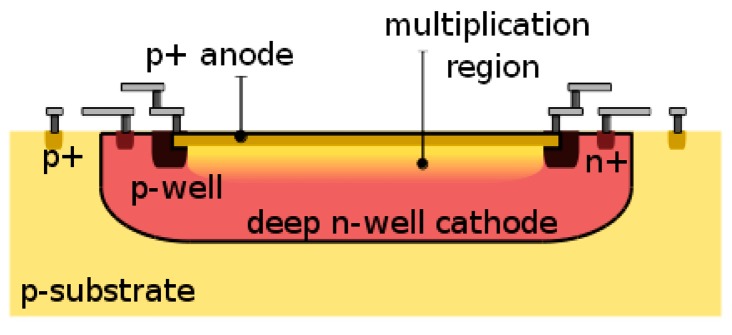
Planar single-photon avalanche diode (SPAD): the p+-n-well junction is biased above breakdown so as to achieve infinite optical gain. A guard ring prevents premature edge breakdown by reducing the electric field in areas at risk, such as the corners of the junction. The deep n-well acts as an insulation mechanism to minimize electrical crosstalk, while optical crosstalk is generally reduced using deep trench isolation (not available in this CMOS technology).

A single-photon avalanche diode (SPAD) is a p-n junction biased above breakdown, so as to operate in Geiger mode. In this design, SPADs are similar to those in [24], comprising a circular p+ active region over n-well, whereas premature edge breakdown is prevented by means of p-well guard rings. Figure 1 shows the cross-section of a planar implementation of a SPAD consistent with CMOS processes. The SPADs are passively quenched, while an active recharge technique is provided [1,2].

The pixel can perform photon counting by means of a one-bit counter, implemented as a static latch. The pixel achieves gated operation by way of three transistors acting as switches. The pixel counter content is transferred to the exterior of the sensor via a fast digital readout channel capable of transferring a complete frame in 6.4 µs. The sensor has a global shutter that gates all the pixels simultaneously for a time as short as 3.8 ns.

**Figure 2:**
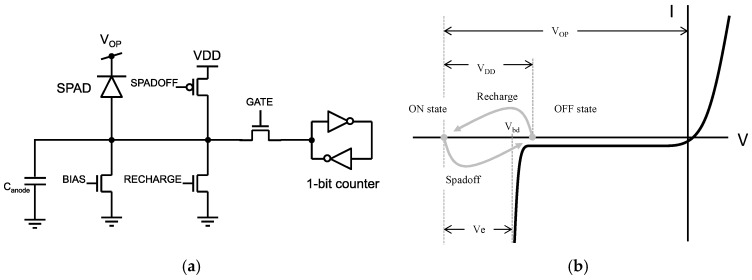
Pixel architecture. (**a**) SPAD configuration with parasitic load (C_anode_); the MOS transistors controlled by signals “SPADOFF”, “GATE”, and “RECHARGE” act as switches that implement gating. The quenching transistor controlled by “BIAS” acts as a non-linear resistor used for quenching. The one-bit counter (represented here as a simplified latch) is used to record photon detection; (**b**) I-V characteristics of the SPAD pixel. In the ON state, the SPAD is biased above breakdown (V_bd_) by a voltage known as excess bias (V_e_). When “SPADOFF” is activated, the SPAD bias is pushed below breakdown; “GATE” is deactivated to isolate the counter; this is the OFF state. To bring the SPAD back to the ON state, “GATE“ is activated, “SPADOFF” is deactivated, and “RECHARGE” is activated for a short time, typically nanoseconds, thereby bringing the bias to the initial state above breakdown. Photon detection triggers a similar cycle with a resulting change of state of the one-bit counter from “L” to “H”.

The pixel conceptual diagram is shown in Figure 2a. The MOS switch “SPADOFF” is activated to bring the SPAD below breakdown, thereby quenching any ongoing avalanche and preventing any future avalanche in the same frame (see Figure 2b). The second MOS switch, “RECHARGE”, is designed to bring the bias voltage close to ground, thereby rapidly recharging the SPAD to its idle bias. This action reactivates the SPAD and, to avoid direct conduction from VDD to ground, it should never be performed simultaneously to “SPADOFF”. The last MOS switch, “GATE”, is used to prevent the one-bit counter from being accidentally set during the gating operations.

**Figure 3:**
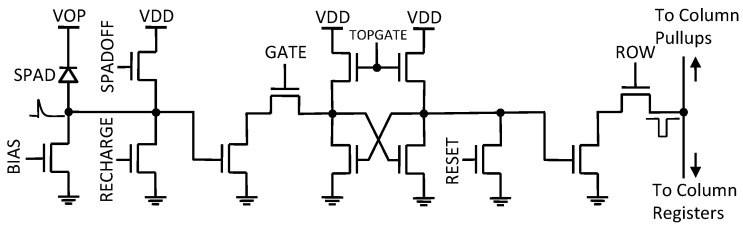
Pixel schematic. The counter is implemented as a latch, which is reset by “RESET” and biased by “TOPGATE”. The content of the counter is read out using a rolling shutter mechanism by setting “ROW” to “H”; when the counter has recorded a photon, a pull-down transistor sets the column to “L” and this state is transferred to a latch at the bottom of the column (not shown in the figure) and then to a PAD via a multiplexer for external processing. A pull-up ensures return of the column to “H” state when no photons are detected. “RECHARGE” and “SPADOFF” are used to turn on and off the SPAD, while “GATE” is used as a pass gate and “BIAS” determines the equivalent resistance used as ballast.

The actual implementation of the pixel is shown in Figure 3. The recharging transistor is controlled by a global “RECHARGE” signal. The switches are implemented as NMOS transistors, while the latch is implemented by way of four NMOS transistors, connected as back-to-back NMOS inverters to eliminate the need for PMOS transistors. The pull-up transistors are critical to control power consumption in the latch during idle phases and settling time during set/reset phases. These transistors can be controlled using an external voltage, “TOPGATE”. The column pull-up transistor is biased so as to minimize the power required to bring the column to ‘L’ while ensuring a readout cycle of 6.4 µs/128 = 50 ns.

The pixel content is stored in a latch at the bottom of the column (not shown in the figure) that stores its value for 50 ns while the other three columns are multiplexed out to the external PAD. A 4:1 multiplexer serializes the output of the latches of four columns to the PADs; it is operated at four times that speed, i.e., 4/50 ns = 80 MHz, which is the maximal operating speed of the PADs in this technology and represents a good speed-power tradeoff.

**Figure 4:**
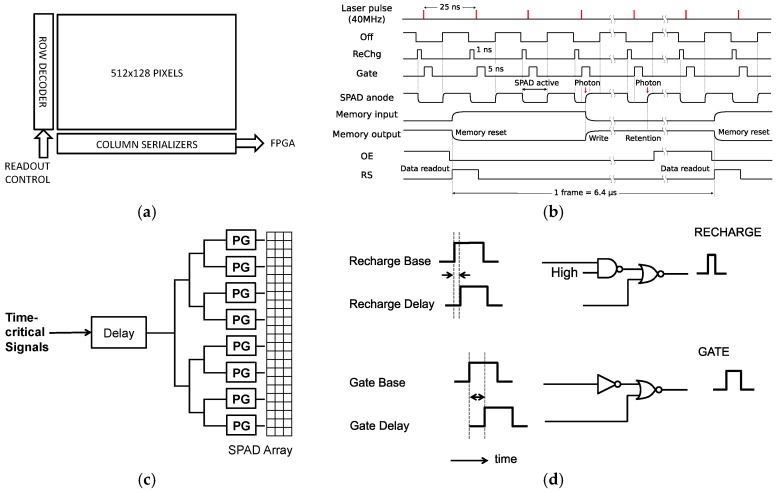
Block diagram of the sensor: (**a**) overall block diagram; (**b**) timing diagram; (**c**) balanced tree network for time-critical signal distribution; (**d**) pulse generation mechanism for in situ generation of accurate pulses insensitive to rise/fall time asymmetries.

The block diagram of the sensor, known as SwissSPAD, is shown in Figure 4a. The timing diagram for the pixel is shown in Figure 4b. The chip features a balanced network that distributes a low-skew version of signals “SPADOFF”, “RECHARGE”, and “GATE” (Figure 4c). Due to their nanosecond length, “RECHARGE”, and “GATE” are distributed as three precisely timed rising-edge signals that are recomposed in situ by means of a pulse generator (PG) shown in Figure 4d.

The timing diagram shows a typical readout cycle, wherein a memory reset is performed at the beginning of the cycle and a series of gating operations follows, until the next readout is performed. The gates are spaced an arbitrary time period (25 ns in this example) and are generally synchronized with a light source. This is done to maximize the effective spatio-temporal fill factor when a fast but dim response is expected from a pulsed light source, as, for example in FLIM. Fewer gates or even a single gate is possible, however, it results in an effective spatio-temporal *FF* computed as

(1) $$FF=FF_{G}\cdot DC=FF_{G}\frac{N\cdot t_{GATE}}{T_{FRAME}}=FF_{G}\cdot f_{GATE}\cdot t_{GATE}$$

where *FF*_G_ is the geometric fill factor, *DC* the duty cycle of gating, *N* the number of gates in a frame, *t_GATE_* the time length of the gate, *T_FRAME_* the period of the frame, and *f_GATE_* the frequency of the gate. Note that even though we reduce the temporal *FF*, light will usually impinge within the gate synchronized with a laser, while the DCR will be reduced. The duty cycle is generally selected to be a fraction of the lifetime of a fluorophore. The delay between gate and excitation light is varied a minimum of 20 ps and a maximum of *T_FRAME_*/*N*, so as to scan the entire laser period 1/*f_GATE_*. *N* is chosen to minimize the pile-up effect. Since the counter only counts, at most, one event, no accumulation is possible during a frame but only digitally after multiple frames. This enables us to construct gray levels in images at the expense of a reduced frame rate [22].

## 3. Binary Pixels

SwissSPAD is an all-digital, clock-driven sensor comprising pixels that can only detect one photon in a frame: we call these pixels “binary pixels”. Photons impinge a binary pixel with an expected arrival rate χ (photons per second) and are distributed in time following a Poisson distribution (the probability of *k* counts per second is $p\left(cps=k\right)=\frac{\chi^{k}e^{-\chi }}{k!}$). Thus, the probability of detecting one or more photons per second is $p\;\left(cps>0\right)=1-e^{-\chi }$. For a non-unity photon detection probability (PDP) and non-zero *FF*, the probability of photon detection per frame $p\left(cpf>0\right)=1-e^{-\chi \cdot PDP\cdot FF\cdot T_{FRAME}}$. The expected photon counts per second measured in the pixel will thus become [25]

(2) $$E\left(C_{M}\right)=\frac{1-e^{-\chi \cdot PDP\cdot FF\cdot T_{FRAME}}}{T_{FRAME}}$$

where C_M_ is the measured SPAD count rate. Thus, even if one photon per frame is expected to impinge on the pixel, the pixel will detect it, on average, a fraction of the time, i.e., it will detect a fraction of a count, on average, per frame. Dark noise in a SPAD is dominated by three sources: thermal (trap-assisted and tunneling), noise, and afterpulsing. Assuming a large dead time, afterpulsing can be ignored and thus, with the exception of hot pixels, most exhibit a noise approaching Poisson statistics. The rate of occurrence of this noise is quantified by dark count rate (DCR). Thanks to its Poissonian nature, DCR is added to the equation as follows

(3) $$E\left(C_{M}\right)=\frac{1-e^{-\left(\chi \cdot PDP\cdot FF+DCR\right)\cdot T_{FRAME}}}{T_{FRAME}}$$

From this equation, one can derive the correction factor for the expected detected SPAD count rate $E\left(C_{D}\right)=\chi \cdot PDP\cdot FF+DCR$, by simply solving the equation w.r.t E(*C*_D_), as follows

(4) $$C_{D}\approx \frac{-\ln\left(1-C_{M}\cdot T_{FRAME}\right)}{T_{FRAME}}$$

Note that E(*C_M_*) and E(*C_D_*) were replaced by *C*_M_, and *C*_D_, respectively, since it is assumed that the correction is applied to a single sample generated by the detector and not the expected value achieved over a very large number of measurements. As can be seen from the equation, this correction is only needed for high values of *C_M_*, above 15 kcps.

**Figure 5:**
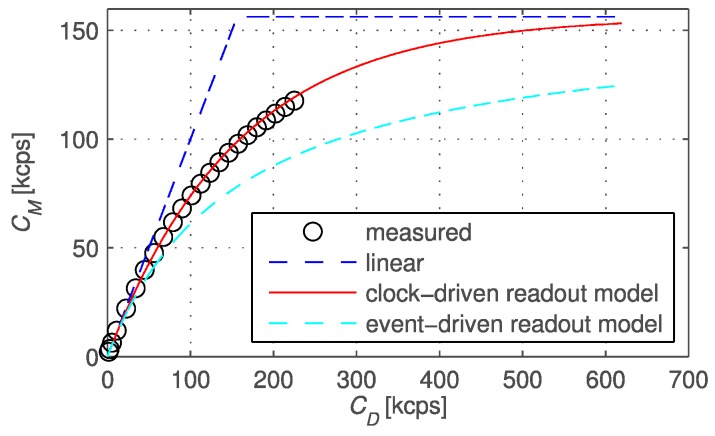
Theoretically predicted and actually recorded (measured) photons in all-digital image sensors as a function of impinging photons, as they are detected and multiplied in a binary pixel. The plot shows clock-driven and event-driven modes in comparison with a non-binary pixel.

However, in this condition, the asymptotic behavior can be used to extend the dynamic range of the pixel, as has been known in the silicon photomultiplier community for several years [26] and in the radiation community from the 1970s [27]. This can be done both in time and in space, whenever multiple pixels are added to make a larger one [28,29]. Figure 5 shows the theoretical and measured response of a binary pixel in clock-driven and in event-driven modes, as compared to the linear response of a non-binary pixel. In clock-driven mode, SPAD recharge or memory reset is applied periodically, asynchronously with respect to SPAD activity, while in event-driven mode recharge is done *T_dead_* after a SPAD avalanche, thus synchronously with SPAD activity. While clock-driven resets at high frequency are not used in single SPAD devices because of possible afterpulsing, arrays with long *T_dead_* do not show increased afterpulsing. Recent work is indicating a trend towards higher pixel resolution and advanced processing [30,31].

## 4. Sensor Fabrication

**Figure 6:**
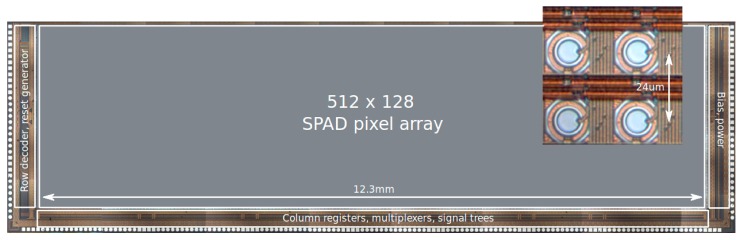
Photomicrograph of the sensor; it was fabricated in a 0.35 µm CMOS process with a pitch of 24 µm. The inset shows a detail of four pixels achieving a fill factor of 5%.

**Figure 7:**
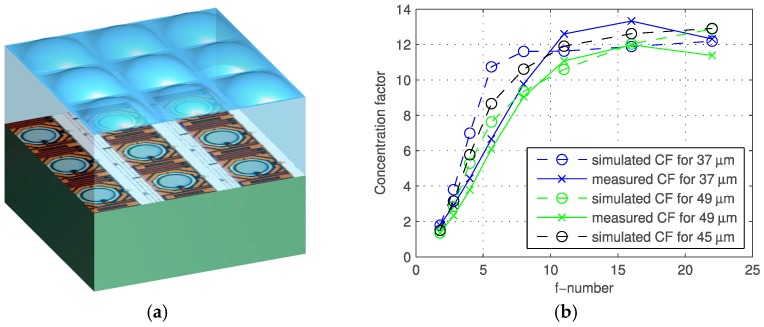
(**a**) Artist’s rendering of the microlens array deposited on the sensor (Courtesy: Juan Mata Pavia); (**b**) measured and simulated concentration factors as a function of the f-number in the main lens [25]. The graph shows simulated and measured data for chips with 37, 45, and 49 μm microlens height.

The sensor microphotograph is shown in Figure 6; the inset shows a detail of the pixels. An array of microlenses (CSEM, Basel, Switzerland) was deposited on the chip matching the pixel pitch to improve light collection through light concentration [32,33]. The microlens array, shown in an artist’s rendering in Figure 7a [34], was measured and simulated as a function of the f-number of the main objective lens, yielding the plot of Figure 7b.

**Figure 8:**
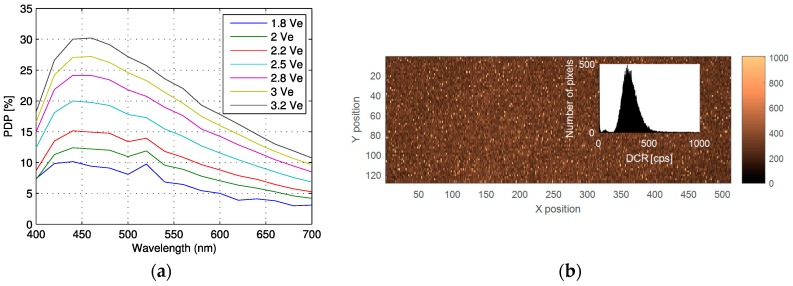
Pixel characterization: (**a**) PDP vs. wavelength at given excess bias voltages; (**b**) DCR distribution at 40 °C for V_e_ = 4.5 V, with an average of 1169 cps and a median of 302 cps. In the inset the DCR distribution is shown.

Thus, the effective fill factor achieved with a lens of f/10 was 60% with high reliability and reproducibility over the entire array. The pixel PDP and DCR are plotted in Figure 8 at room temperature.

The sensor was also characterized in terms of afterpulsing. The measurement was achieved by means of the inter-arrival response method introduced in [35]. The pixels were exposed to a uniform wide-spectrum light source and the inter-arrival time of the response was stored in the FPGA for an integration time of 80 s. A histogram was then constructed confirming the exponential behavior of the response due to the Poissonian nature impinging photons. Afterpulsing probability *APP*(*t*) is approximated as

(5) $$APP\left(t\right)\approx \frac{\int_{t}^{\infty }\left[h_{M}\left(\tau \right)-h_{F}\left(\tau \right)\right]d\tau }{\int_{0}^{\infty }h_{M}\left(\tau \right)d\tau },\;\;t>t_{DT}$$

**Figure 9:**
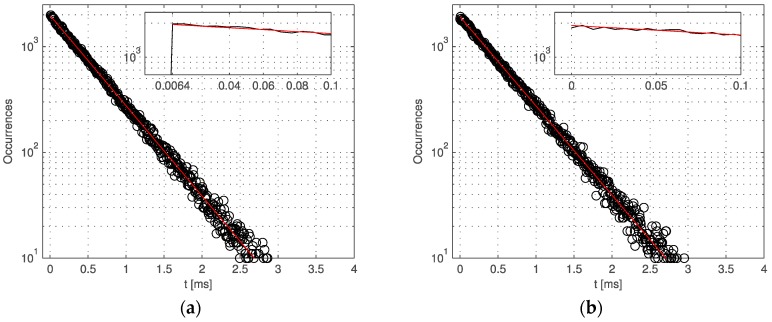
Afterpulsing and crosstalk: (**a**) inter-arrival histogram showing no measurable afterpulsing above the dead time of 6.4 µs, the inset shows a zoom of the plot; (**b**) crosstalk measured using the same method. Note that in this case the dead time is zero, given by the fact that two pixels are independently firing upon detection of a photon [25].

In the equation, $h_{M}\left(\tau \right)$ is the measured histogram, $h_{F}\left(\tau \right)$ the exponential fit, and $t_{DT}$ the dead time of the pixel, in this case 6.4 µs. Crosstalk probability, or simply crosstalk, XT(τ) is computed in a similar way, wherein the inter-arrival time is measured between two adjacent pixels, as

(6) $$XT\left(t\right)\approx \frac{\int_{t}^{\infty }\left[h_{ij}\left(\tau \right)-h_{F}\left(\tau \right)\right]d\tau }{\int_{0}^{\infty }h_{ij}\left(\tau \right)d\tau }$$

where $h_{ij}\left(\tau \right)$ is the inter-arrival time histogram measured between pixels *i* and *j*. Afterpulsing and crosstalk are reported in Figure 9a,b, respectively.

## 5. Results

**Figure 10:**
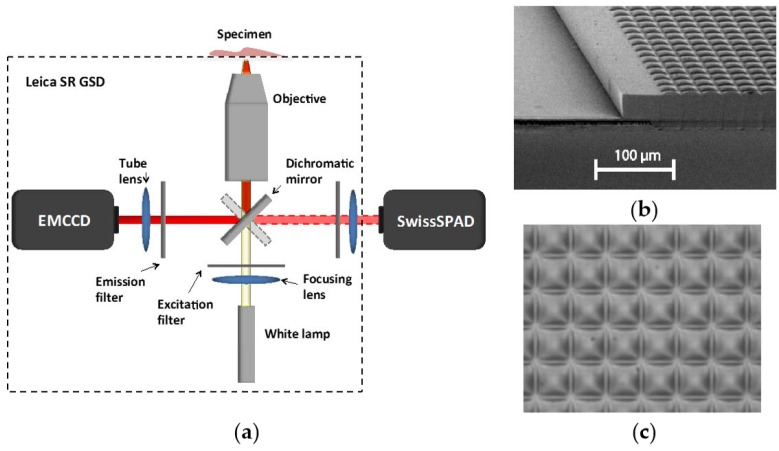
(**a**) Schematic microscope setup used in the imaging experiments based on the Leica SR GSD microscope (Leica Microsystems Wetzlar, Germany); (**b**) SEM scan of the microlens array deposited on the sensor; (**c**) Optical micrograph of the microlens array.

The sensor was used to image a large number of biological samples using fluorescence intensity and fluorescence lifetime imaging microscopy. Fluorescence intensity was achieved using a setup based on a dual port Leica SR GSD super resolution microscope (Leica Microsystems, Wetzlar, Germany) where SwissSPAD and an Andor iXon3 897 BV EMCCD were placed on the two ports of the microscope using the same illumination conditions for comparison purposes (Figure 10). As an illustration, several biological samples are shown hereafter. First, let us consider BPAE cells labeled with MitoTracker Red CMX Ros, Alexa Fluor 488, and DAPI dyes. SwissSPAD was used at V_e_ = 4.5 V. The EMCCD raw intensity image was converted to a photon count image using counts *D* = (*d − b*) × *g*_amp_/*g*_EM_, where *d* is the digital intensity value, *b* the bias offset, *g*_amp_ the preamp gain value and *g*_EM_ the EM gain. Due to pixel size differences, 2 × 2 SPAD pixels and 3 × 3 EMCCD pixels were binned to obtain counts for the same area. MATLAB software was used to find the overlapping area of the two images and compare the intensities.

**Figure 11:**
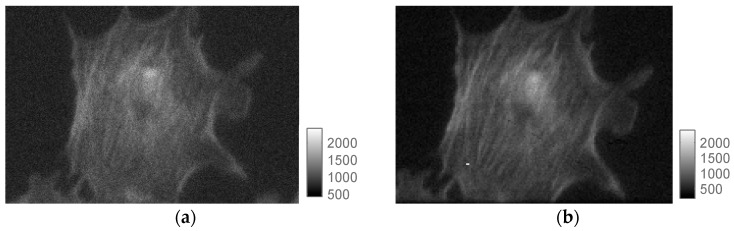
BPAE cell imaging. The cells were labeled with MitoTracker Red CMX Ros, Alexa Fluor 488, and DAPI dyes and imaged in a microscope using two ports matching the photon counts per pixel in each sensor. (**a**) shows the EMCCD and (**b**) the SwissSPAD images obtained with 10 ms and 73.4 ms exposure, respectively. The scales indicate photon counts per frame.

**Figure 12:**
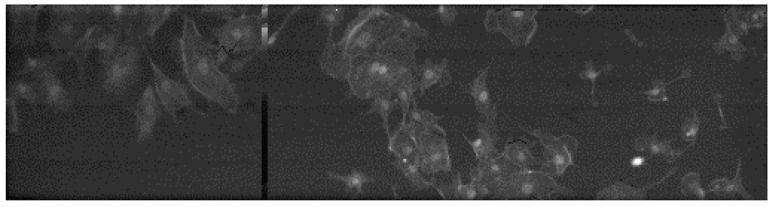
Widefield fluorescence imaging of BPEA QGS cellular clusters labeled with MitoTracker Red CMX Ros, Alexa Fluor 488, and DAPI dyes (magnification = 10×). The dead column in the image is due to a false connection between the imager and the FPGA.

Figure 11 shows the images obtained with the EMCCD (a) and SwissSPAD (b); the exposure times were 10 ms and 73.4 ms, respectively, to match the number of collected photons. The scale shows the number of collected photons per exposure. Figure 12 shows a widefield image of a cellular cluster magnified 10× using the same microscope setup.

**Figure 13:**
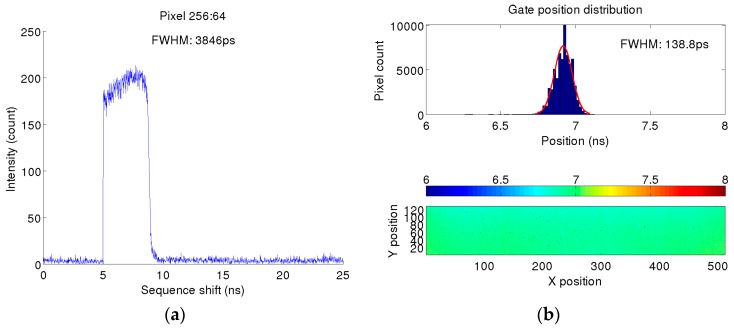
Timing characterization of the gating mechanism: (**a**) characteristic sensitivity at the minimum gate width of 3.8 ns; (**b**) gate position (in time) statistics as a function of pixel position [23].

Fluorescence lifetime images could be obtained by sliding the gate start time from 0 to 10 ns with a step of 20 ps and integrating 255 frames per step, whereas the gate timing performance is essential for high quality FLIM images. The timing performance of the gate is summarized in Figure 13: the response of the sensor is shown in Figure 13a for a random pixel with minimum gate width and the uniformity of its position and length is shown in Figure 13b. Figure 13 shows steep edges of the counting response of the sensor when gating is used, and should not be mistaken with the signal shape of the “Gate” signal. The photosensitive window is defined by the falling edge of “Recharge” and falling edge of “Gate” (if “Gate” occurs after “Recharge”). A large vertical dimension with 128 pixels resulting in 3 mm long metal wires introduces an undesired RC component, limiting the minimal gate width. Although the metal wires of “Gate” and “Recharge” are equal, “Recharge” was designed larger to enable a shorter signal. This introduces a mismatch in “Gate” and “Recharge“ RC, and nonuniformity of photosensitive window widths. Smaller technology nodes will decrease transistor gate capacitances, and wider metal lines through the column can reduce the resistance while keeping the parasitic capacitance dominated by the lateral component constant. The use of repeaters is also an option. Both the metal widening and repeater though can reduce fill factor. A smaller pixel pitch will also reduce the RC component of the line. The right edge of Figure 13a corresponds to the falling edge of the “Recharge” signal and it represents the critical edge for FLIM. A theoretical approach of a FLIM measurement with gating is a convolution between an exponential distribution signal and a rectangular gate signal. The fall time of the falling edge should be small in comparison to the lifetime of the exponential distribution to assure high precision measurements. This fall time is a similar measure as the instrumentation response function (IRF) in TCSPC.

**Figure 14:**
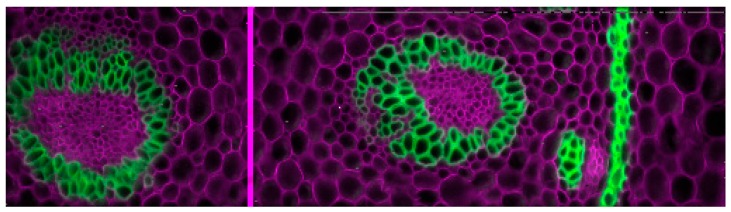
Composite fluorescence image of a thin slice of a plant root stained with a mixture of fluorescent dyes. The picture was obtained with two 20 nm interference filters centered at 530 and 640 nm, respectively. The dead column in the image is due to a false connection between the imager and the FPGA.

Figure 14 reports fluorescence intensity images of samples stained with Safranin and Fast Green, having peak excitation wavelengths of 530 nm and 620 nm, respectively. Filtering was used in two subsequent exposures of the same sample and software based recomposition was then applied. Pictures on Figure 11, Figure 12, and Figure 14 were corrected for DCR and possible count compression using Equation (4).

**Figure 15:**
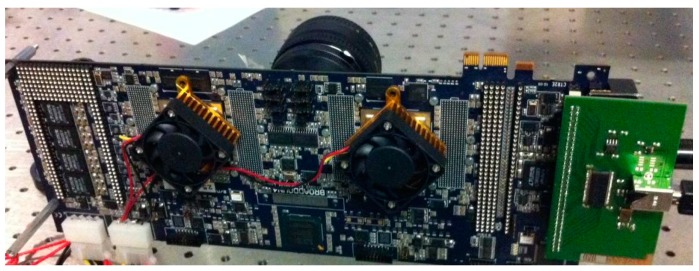
Physical appearance of the camera based on SwissSPAD; a daughterboard hosting the chip is electrically connected to a motherboard hosting two Xilinx-IV that process the raw frames generated by the chip and format them to send them through a USB2 link.

Thanks to the frame readout period of 6.4 µs, one can achieve a maximum frame rate of 156 kfps, whereas a Virtex™ IV or Spartan™ 6 FPGA (Xilinx, San Jose, CA, USA.) is used to acquire and store the one-bit frames. Figure 15 shows the physical appearance of the system, whereas a daughterboard hosting SwissSPAD is electrically connected to a motherboard hosting two Xilinx-IV FPGAs for acquisition and formatting of the data that are then sent to a Mac/PC through USB2 link.

**Figure 16:**
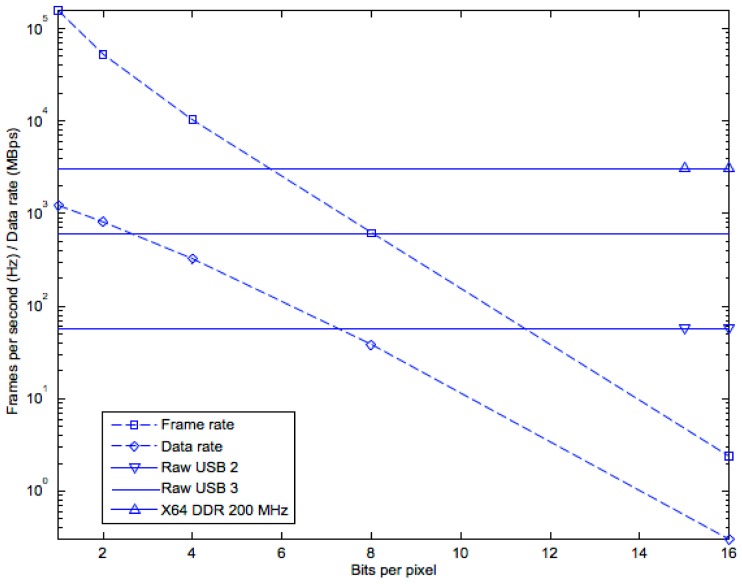
Tradeoff between pixel ENOB and EFPS in SwissSPAD. X64 DDR, USB-2, and USB-3 are shown as horizontal lines to represent maximal supported data rates.

To construct gray level images, the one-bit frames may be accumulated in the FPGA and transferred to the Mac/PC through a USB-2 or USB-3 link; by doubling the number of frames, the pixel intensity resolution increases by one bit [22]. The tradeoff between pixel effective number of bit (ENOB) and effective frames-per-second (EFPS) is shown in Figure 16. While the speed of a DDR memory is high enough to keep up with the data rate generated by the FPGA at any ENOB, USB-2, and USB-3 links to the PC do not allow continuous recording at all ENOBs, making an intermediate memory like DDR2 necessary. An USB-2 link can transfer eight-bit frames continuously.

**Figure 17:**
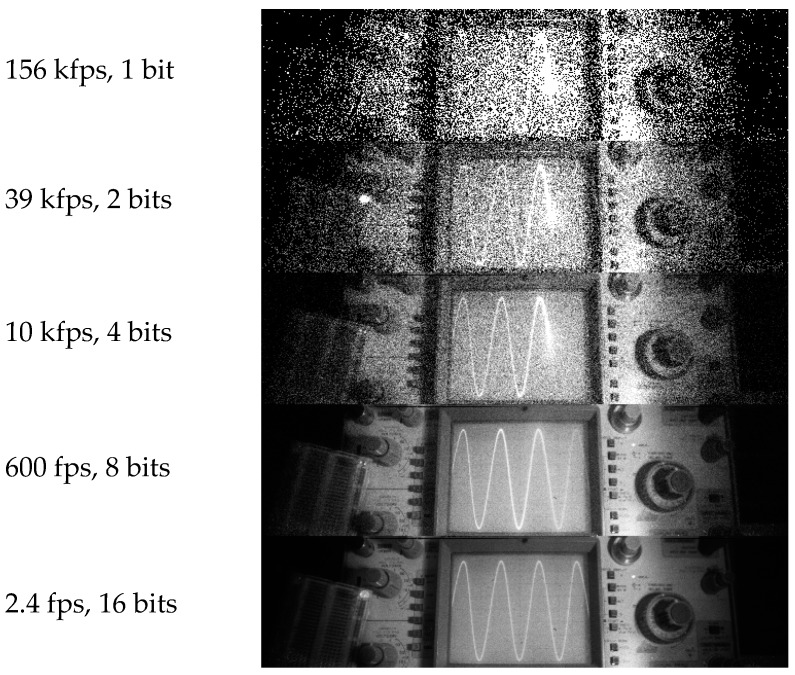
Tradeoff between effective number of bits (ENOB) and frame rate, from 156 kfps to 2.4 fps [23].

The sequence of Figure 17 shows images of an analog oscilloscope obtained without accumulation (156 kfps, 1 bit) and with several levels of accumulation from 4× to 65,536×, resulting in a pixel ENOB of 2 and 16 bits, respectively, during the cumulative frame. Unlike conventional cameras, the pixel ENOB is derived by the simple expression

$$ENOB=\log_{2}\left(N_{pixel}\right)$$

where *N*_pixel_ is the maximum possible number of accumulated counts in the pixel during a cumulative frame. The SNR per pixel, and over the entire sensor, is approximated by

$$SNR_{pixel}=\sqrt{\;N_{pixel}}$$

This approximation assumes no readout noise and lower count rates with linear response, thus a Poisson limited noise at any frame rates and no saturation. The images in Figure 17 show Poisson limited noise in the images at five different exposure times.

**Figure 18:**
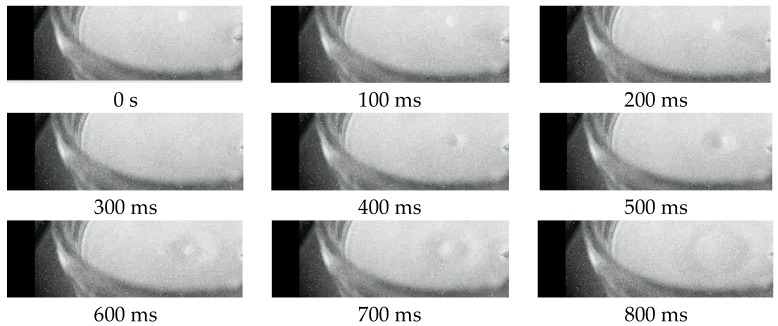
Sequence of a drop fall recorded at 1200 fps; the frame-to-frame time interval shown in the sequence was 100 ms.

In Figure 18a sequence of a fast event is shown; the frame rate was fix at 1200 frames-per-second, of which one frame every 100 ms is depicted. A global shutter was used achieving deep-subnanosecond uncertainty of the gate width and position.

**Figure 19:**
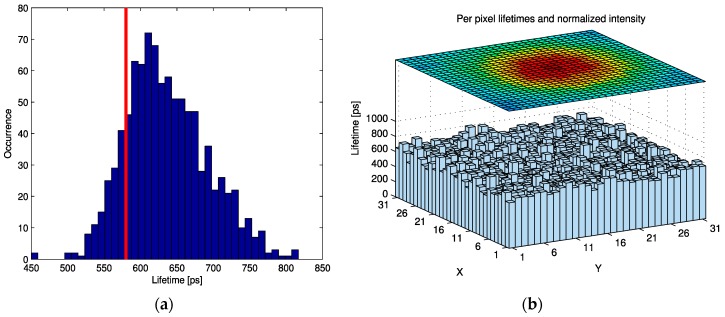
(**a**) FLIM results show extracted lifetimes distribution of 31 × 31 pixels compared to reference lifetime of 40 µM ICG in milk (red). (**b**) shows the comparison of intensity and lifetime per pixel.

The ability of SwissSPAD to acquire lifetime images was demonstrated in a lab setup using point detection. Indocyanine green (ICG) in milk with a concentration of 40 µM was excited using a 790 nm laser with 55 ps pulse width and 100MHz repetition rate synchronized with the SwissSPAD gating. Fluorescence intensity from the excited spot was measured for 512 gate windows offset by a fraction of the repetition period (25 ps). From the response similar to the IRF shown in Figure 13 convolved with an exponential decay the lifetime is extracted by fitting against a set of models constructed from the IRF used in these measurements. Figure 19 shows the per pixel extracted lifetime and normalized intensity over the excited spot. The extracted lifetimes with μ = 636 ps and σ = 56 ps overestimate the 580 ps reference lifetime given in literature [36]. Homulle et al. showed in [37] how the accuracy of lifetime extraction from gated measurements can be improved through refinement of the modeling and simulation.

The sensor specifications are listed in Table 1. All the measurements were performed at room temperature, unless otherwise indicated.

The sensor is currently used by a number of researchers in different institutions under use warranty based on GNU policies.

## 6. Conclusions

We reported on a 512 × 128 SPAD image sensor operating at a maximum speed of 156 kfps with a low-skew global shutter. The sensor has been successfully used in a variety of applications, involving time-resolved capture of fast events. It can be operated in TCSPC mode to achieve, for instance, FLIM images. The sensor is highly versatile and allows one to achieve tradeoffs between frame speed and resolution. Thanks to its zero readout noise, the sensor noise is always Poisson limited, thus enabling investigations in photon starved regimes. Future work includes extensive modeling for super-resolution microscopy and the design of a larger array with reduced DCR and increased PDE. 

## Figures and Tables

**Table 1:** Specifications of the sensor SwissSPAD. All the values are reported at room temperature.

Parameter	Value	Condition
Peak PDE	20%	450 nm
Max. frame rate	156 kfps	1 bit ENOB ^1^
Readout noise	0 cps	
Dark counts	200 cps	25 °C
Pixel pitch	24 µm	
Active area	28.3 µm^2^	Drawn area
Imager size	12.3 × 3.1 mm^2^	
Operating temperature	25 °C	
Afterpulsing probability	0.3%	1 µs dead time
Crosstalk	<0.3%	
PRNU	<1.8% ^2^	
Min. gate width	3.8 ns	
Gate skew	<150 ps	Sigma

^1^ ENOB denotes effective number of bits for the pixel resolution. ^2^ PRNU denotes photo-response non-uniformity without compensation.

## Supplementary Materials

The full videos are available online at http://aqua.epfl.ch and http://www.aqua.ewi.tudelft.nl/movies/.

## Abbreviations

The following abbreviations are used in this manuscript:

SPAD	Single-photon avalanche diode
FLIM	Fluorescence lifetime imaging microscopy
EMCCD	Electromultiplied charge-coupled device
SNR	Signal-to-noise ratio
PRNU	Photo-response non-uniformity
DCR	Dark count rate
PDP	Photon detection probability
PDE	Photon detection efficiency
BPAE	Bovine pulmonary artery endothelial cells
DAPI	4′,6-Diamidino-2-phenylindole
IRF	Instrumentation response function
TCSPC	Time-correlated single photon counting
ENOB	Effective number of bits
ICG	Indocyanine green
